# Prefrontal Inter-brain Synchronization Reflects Convergence and Divergence of Flow Dynamics in Collaborative Learning: A Pilot Study

**DOI:** 10.3389/fnrgo.2021.686596

**Published:** 2021-06-03

**Authors:** Takayuki Nozawa, Mutsumi Kondo, Reiko Yamamoto, Hyeonjeong Jeong, Shigeyuki Ikeda, Kohei Sakaki, Yoshihiro Miyake, Yasushige Ishikawa, Ryuta Kawashima

**Affiliations:** ^1^Institute of Development, Aging and Cancer, Tohoku University, Sendai, Japan; ^2^Research Institute for the Earth Inclusive Sensing, Tokyo Institute of Technology, Tokyo, Japan; ^3^Department of British and American Studies, Kyoto University of Foreign Studies, Kyoto, Japan; ^4^Graduate School of International Cultural Studies, Tohoku University, Sendai, Japan; ^5^RIKEN Center for Advanced Intelligence Project, Tokyo, Japan; ^6^Department of Computer Science, Tokyo Institute of Technology, Yokohama, Japan

**Keywords:** shared flow, inter-brain synchronization, fNIRS hyperscanning, collaborative learning, engagement, dynamics

## Abstract

Flow is a highly motivated and affectively positive state in which a person is deeply engaged in an activity and feeling enjoyment from it. In collaborative activities, it would be optimal if all participants were in a state of flow. However, flow states fluctuate amongst individuals due to differences in the dynamics of motivation and cognition. To explore the possibility that inter-brain synchronization can provide a quantitative measure of the convergence and divergence of collective motivational dynamics, we conducted a pilot study to investigate the relationship between inter-brain synchronization and the interpersonal similarity of flow state dynamics during the collaborative learning process. In two English as a Foreign Language (EFL) classes, students were divided into groups of three-four and seated at desks facing each other while conducting a 60-min group work. In both classes, two groups with four members were randomly selected, and their medial prefrontal neural activities were measured simultaneously using wireless functional near-infrared spectroscopy (fNIRS) devices. Later the participants observed their own activities on recorded videos and retrospectively rated their subjective degree of flow state on a seven-point scale for each 2-min period. For the pairs of students whose neural activities were measured, the similarity of their flow experience dynamics was evaluated by the temporal correlation between their flow ratings. Prefrontal inter-brain synchronization of the same student pairs during group work was evaluated using wavelet transform coherence. Statistical analyses revealed that: (1) flow dynamics were significantly more similar for the student pairs within the same group compared to the pairs of students assigned across different groups; (2) prefrontal inter-brain synchronization in the relatively short time scale (9.3–13.9 s) was significantly higher for the within-group pairs than for the cross-group pairs; and (3) the prefrontal inter-brain synchronization at the same short time scale was significantly and positively correlated with the similarity of flow dynamics, even after controlling for the effects of within- vs. cross-group pair types from the two variables. These suggest that inter-brain synchronization can indeed provide a quantitative measure for converging and diverging collective motivational dynamics during collaborative learning, with higher inter-brain synchronization corresponding to a more convergent flow experience.

## Introduction

In psychology, flow is a state of deeply and energetically engaging in and enjoying an activity at hand (Csikszentmihalyi, 1997). People generally experience flow when: (1) the perceived challenge of an activity is high and in balance with their own capacities or skills; (2) they receive immediate feedback about how well they are doing; and (3) the goal is clear. Flow is also linked to intrinsic motivation, which is instrumental in achieving various goals (Deci and Ryan, 2000). In education, in addition to the challenge-skill balance, the experience of flow is promoted by active participation rather than passive reception of information (Shernoff et al., 2003). Flow experience in learning can also improve a students' psychological well-being, perceived learning, satisfaction, and learning performance (Rossin et al., 2009; Steele and Fullagar, 2009; Landhäußer and Keller, 2012; Buil et al., 2019).

In group learning, such as in classrooms, teachers find it optimal when the entire class functions together in the state of flow, being in synch with one another (Kent, 2013). However, the degree of flow fluctuates from moment to moment and varies among learners. The cause of divergent flow dynamics between learners can be due to some students being distracted, not sufficiently engaged, or perceiving the task at hand to be too difficult or too easy for their capabilities. Whatever the cause, if convergence and divergence of flow experience between learners can be detected using an objective measure, it may help to achieve optimal educational situations. For example, if a detection system identifies a student in a collaborative learning project who diverges from the other group members in terms of their flow state, the teacher can be alerted to instigate countermeasures, such as guiding the student to focus more, or changing the group composition to make the members' capability levels more compatible with one another. Alternatively, giving feedback on shared flow dynamics to the learners themselves could facilitate behavioral changes that lead to better collaborations.

Recently, the hyperscanning technique, in which the brain activity of multiple people is simultaneously recorded, has been increasingly applied to objectively characterize a wide range of social interactions (Nam et al., 2020). Hyperscanning studies on natural verbal communications have shown that inter-brain synchronization between communication partners reflects various aspects of communication, such as the existence of face-to-face communication (Jiang et al., 2012; Nozawa et al., 2016), emergence of leader-follower relationships (Jiang et al., 2015), and agreement vs. disagreement (Hirsch et al., 2021). Specifically, studies on real educational classroom settings using electroencephalography (EEG) and functional near-infrared spectroscopy (fNIRS) hyperscanning have shown that inter-brain synchronization is associated with higher engagement amongst students (Yamamoto et al., 2015; Dikker et al., 2017; Bevilacqua et al., 2018; Brockington et al., 2018). Based on these results, we hypothesized that the convergence and divergence of dynamically changing flow states between learners could also be associated with inter-brain synchronization between learners, with those pairs who share flow dynamics showing higher inter-brain synchronization.

To test our hypothesis, we applied fNIRS hyperscanning to real active learning activities held in English as a Foreign Language (EFL) class at a university in Japan, and investigated the relationship between the similarity of flow dynamics between learners and their medial prefrontal inter-brain synchronization. The medial prefrontal cortex (mPFC) has been implicated in social cognition and communication (Gilbert et al., 2006; Suda et al., 2010), and inter-brain synchronization in the mPFC has been shown to reflect different communication modes and quality (Nozawa et al., 2016; Liu et al., 2019). In addition, the mPFC is easily accessible for the wireless fNIRS recording (see Methods). We targeted a collaborative group learning setting because group dynamics and shared engagement are especially important in collaborative learning (Järvelä et al., 2010). Furthermore, the division into groups enabled us to compare within-group and cross-group learning pairs. This approach allowed us to test the following hypotheses: (1) flow dynamics are more convergent (i.e., temporally more correlated) between members of the same group who work together than between learners belonging to different groups; (2) inter-brain synchronization is higher between group members, who collaborate with each other, than between cross-group learners who share the same class but do not directly collaborate; and (3) the higher the inter-brain synchronization between a pair of learners, the more similar their dynamics of experienced flow would be; thus, inter-brain synchronization would provide an objective indicator of shared flow dynamics.

## Materials and Methods

### Ethics Statement

This study was approved by the Ethics Committee of Tohoku University Graduate School of Medicine and was conducted in accordance with the Declaration of Helsinki. All participants were briefed on the experimental procedure and provided written informed consent prior to participating in the experiments.

### Participants

Two English as foreign language (EFL) active learning classes at the Kyoto University of Foreign Studies, Japan, participated in this study. The two classes consisted of 1st- or 2nd-grade students (27 in one class and 29 in the other). Among them, 16 students (age 19–22 years; 11 females and five males) were subjected to the fNIRS measurement and the retrospective evaluation of flow dynamics (see section fNIRS Brain Activity Measurements).

### Experimental Procedures

**Figure 1:**
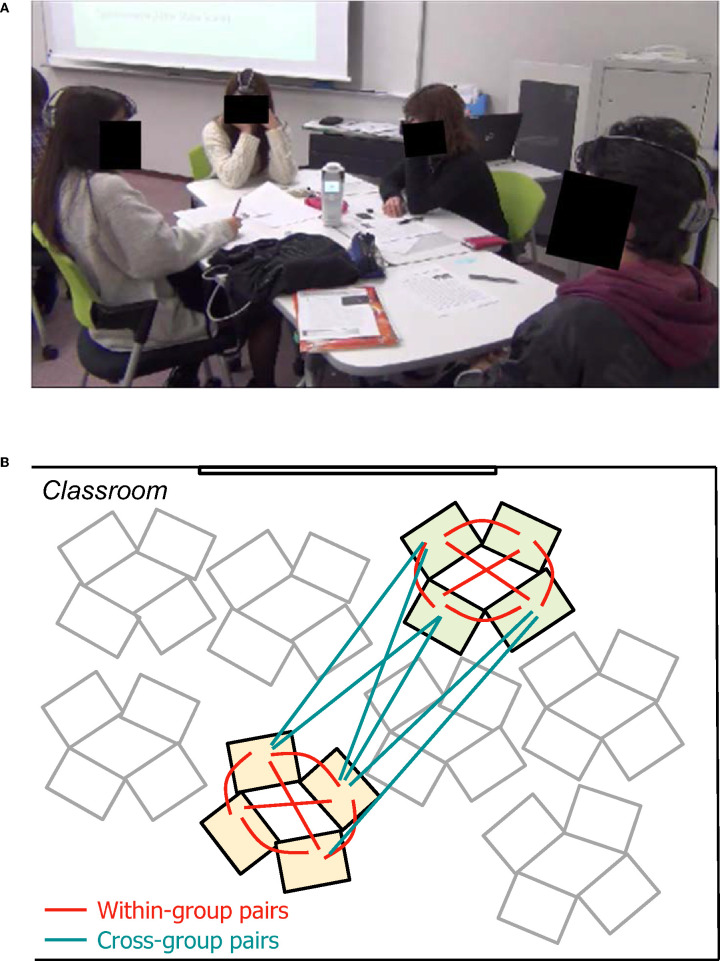
**(A)** Snapshot of a working collaborative learning group with their mPFC activities being measured with wireless fNIRS devices. **(B)** Illustration of student pair types. Within-group pairs and cross-group pairs are indicated in red and blue lines, respectively. For visibility, only six out of 16 cross-group pairs are illustrated.

In each class, students were divided into groups of four or three members and seated at desks facing each other (Figure 1A). In the preparation phase, students were asked to read and understand the script of a speech by Steve Jobs. They then watched a video of the actual speech given by Steve Jobs to deepen their understanding of their reading. Subsequently, they conducted a 60-min group study session, which consisted of four group work activities (GW1-4): GW1 “Connecting the Dots,” they picked out important events from Jobs' personal history and created connected dots of his life; GW2 “Reading a supplemental article,” in turn they read aloud a supplemental text on college dropout in the U.S.; GW3 “Linking Readings One and Two,” they synthesized information from the two readings and completed a worksheet together; and GW4 “Evaluative Discussion,” they discussed college dropout in Japan, comparing different opinions and making judgments. The task switch timings between the four group activities were the same across the groups in each class, but the pacing within each activity was decided by each group. The group work activities were video-recorded for the later retrospective evaluation (see section Retrospective Evaluation of Subjective Flow Dynamics).

### fNIRS Brain Activity Measurements

In each of the two classes, we randomly selected two groups of four members and simultaneously recorded their brain activities throughout the group work. Gender constitution of the four groups were: four females; three females and one male; three females and one male; and one female and three females. We measured the neural activities of the mPFC in eight students using a wireless continuous-wave fNIRS device (Yamamoto et al., 2015; Nozawa et al., 2016; Ikeda et al., 2017). The fNIRS device is lightweight (100 g including battery), comfort-to-wear, and enables the simultaneous measurement of prefrontal neural activities of interacting people. The optode component of the device contains one infrared light source (wavelength 810 nm) and two light detectors at a distance of 1.0 and 3.0 cm from the light source. Using the modified Beer-Lambert law (Delpy et al., 1988; Scholkmann et al., 2014), changes in the detected light intensities were converted to the concentration changes of total hemoglobin on the optical path of the source-detector. Signals were sampled at a frequency of 10 Hz and transmitted to a host computer using the ZigBee wireless network protocol. This system enables the simultaneous measurement of up to 20 individuals. See Nozawa et al. (2016) for more details on the fNIRS device.

The placement of the fNIRS device followed that of a previous study (Nozawa et al., 2016). The center of the optode component was positioned at the center between FP1 and FP2 according to the international 10-20 system for EEG electrode placement, which covers the rostral limit of the superior frontal gyrus (Homan et al., 1987). The light source was placed on the subject's left-hand side, a light detector with a 3 cm distance from the source was on the right-hand side, and a light detector with a 1 cm distance from the source was placed between them. Any makeup on the measurement position was removed and hair brushed away to ensure good optode-skin contact. The optode component was covered with black rubber to shield it from external light. The students were instructed to adopt and maintain a relaxed posture in the seat, and to avoid rapid head movements as much as possible.

### Retrospective Evaluation of Subjective Flow Dynamics

The 16 students who were subjected to the fNIRS recording conducted a retrospective evaluation of their subjective flow dynamics. They were first explained about the concept of flow, based on the description by Csikszentmihalyi (1997), with flow described as “a state in which you are so involved in the activity at hand that nothing else seems to matter. The experience itself is so enjoyable that you will continue the activity for the sheer sake of doing it. In this state, you simultaneously experience concentration, interest, and enjoyment. It is also referred to as “being in the zone.”” Then, watching the video recording of themselves performing the group work, they retrospectively rated their flow level for every 2-min segment on a seven-level scale from 1 (“very low”) through 4 (“neutral”) to 7 (“very high”). This resulted in a series of 30 time points of flow level for the 60-min group work.

### Analyses of Flow Dynamics Similarity and Inter-Brain Synchronization

We calculated the similarity of flow dynamics and prefrontal inter-brain synchronization for all possible pairs of eight students in each class (Figure 1B). Thus, we had 24 pairs (6 pairs/group × 2 groups/class × 2 classes) of students in the same group (“within-group pairs”) and 32 pairs (4 × 4 pairs/class × 2 classes) of students who belonged to different groups (“cross-group pairs”).

The similarity of the flow dynamics of each student pair was evaluated using the Pearson correlation of their flow-level time series. To enhance the normality of the distribution of the sample correlation coefficients, Fisher z-transformation *z* = *arctanh*(*r*) was applied to the coefficients. Then, the flow dynamics correlation values were compared between the pair types to test hypothesis (1) that the flow dynamics are more convergent (i.e., correlation is higher) for the within-group pairs than for the cross-group pairs. Taking into account the dependence structure in the pairwise similarity data, we used a non-parametric permutation test to evaluate the significance of difference between the pair types (Pesarin and Salmaso, 2010; Winkler et al., 2015). First, all possible random permutations re-assigning the eight students into two groups within each class were generated and combined for the two classes. Then, the difference of mean similarity values between the within-group and cross-group pairs for each instance of permutation was gathered to generate the null distribution. Finally, the *p*-value of the observed difference of means was calculated as the proportion of permutation-based differences at least as extreme as the observed difference value. Furthermore, the correlation values were also used to test the relationship between shared flow dynamics and prefrontal inter-brain synchronization (see below).

The fNIRS signals were preprocessed using MATLAB (MathWorks Inc.). First, the drift components were removed from each signal using detrending. Second, wavelet-based motion artifact reduction (Molavi and Dumont, 2012; Brigadoi et al., 2014), implemented as “hmrMotionCorrectWavelet” function in the HomER2 toolbox (Huppert et al., 2009), was applied to correct for spike-like artifacts. Then, dual source-detector regression (Saager et al., 2011) was applied to regress out the shallow-tissue signal component, which is dominated by non-neuronal systemic and motion-related noises and can be captured by the 1 cm source-detector channel (Fukui et al., 2003; Strangman et al., 2014), from the 3 cm source-detector signal that contains both the non-neuronal shallow and brain-originated deep components, thus extracting the brain-originated component.

With the neural signals extracted through the preprocessing steps above, the prefrontal inter-brain synchronization for each pair of students was calculated using wavelet transform coherence (WTC) (Torrence and Compo, 1998; Grinsted et al., 2004). WTC evaluates a localized correlation coefficient in time-frequency space and is thus suitable for capturing transient and bi-directional synchronization, which is expected in unstructured naturalistic communications involving multiple individuals, such as collaborative learning settings. WTC has been used in many fNIRS hyperscanning studies to evaluate inter-brain synchronization (Scholkmann et al., 2013; Minagawa et al., 2018). For the computation, the cross wavelet and wavelet coherence toolbox (http://grinsted.github.io/wavelet-coherence/) was used.

For each student pair, inter-brain synchronization at each Fourier period (inverse of Fourier frequency) was temporally averaged over the entire duration of the group activities, except for the time points within the cone of influence to avoid any contaminating influences from edge effects (Torrence and Compo, 1998). Cerebral blood flow signals in the time scales of the so-called low-frequency oscillations (0.01–0.198 Hz) have been shown to produce more reliable estimates of within-brain neuronal synchronization than higher frequencies (Zuo et al., 2010). Furthermore, fNIRS signals in shorter time scales are known to be susceptible to cardiac pulsatory (0.8–2.5 Hz) and respiratory (0.15–0.3 Hz) noise components (Matthews et al., 2008; Lu et al., 2010). Thus, we focused on inter-brain synchronization in the period range of 6.7–100 s (equivalent to 0.01–0.15 Hz). The temporally averaged inter-brain synchronization values at each period in this range were subjected to the same permutation method as above to test hypothesis (2) that the prefrontal inter-brain synchronization is higher for within-group pairs than for cross-group pairs. False discovery rate (FDR) adjustment (Benjamini and Hochberg, 1995) was applied for multiple testing of the periods, and the test results were regarded as significant with q < 0.05.

To test hypothesis (3) that higher prefrontal inter-brain synchronization is positively associated with more similar flow dynamics, we conducted a correlation analysis. Based on the test of hypothesis (2) above, we identified the period range in which inter-brain synchronization was significantly higher for within-group than for cross-group pairs (thus sensitive to the collaborative interaction) as the period of interest. The inter-brain synchronization values in the period of interest were averaged for each pair of students and correlated with the similarity values of flow dynamics. A non-parametric permutation method was used to evaluate the significance (*p*-value) of the correlation. We generated 10,000 random permutations shuffling the students in each class. Each permutation of subjects produces a possible permutation of pairs. We tested the significance of the observed correlation against the null distribution of correlation values between the original inter-brain synchronization values and the flow dynamics similarities with the permutated pair labels. Furthermore, we also tested the correlation between inter-brain synchronization and flow dynamics after controlling for the effects of within- and cross-group pair types from the two variables (i.e., partial correlation).

We additionally evaluated the interpersonal synchronization of the non-neuronal shallow signals that were obtained from the 1 cm source-detector channel and subjected to the same preprocessing procedure, except for the dual source-detector regression. Then, for this interpersonal shallow signal synchronization, we repeated the same analyses corresponding to the testing of hypotheses (2) and (3) above. The comparison of the results with inter-brain synchronization and with interpersonal shallow signal synchronization helped us to check whether the obtained inter-brain synchronization results were indeed of neural origin.

## Results

### Subjective Flow Dynamics

**Figure 2:**
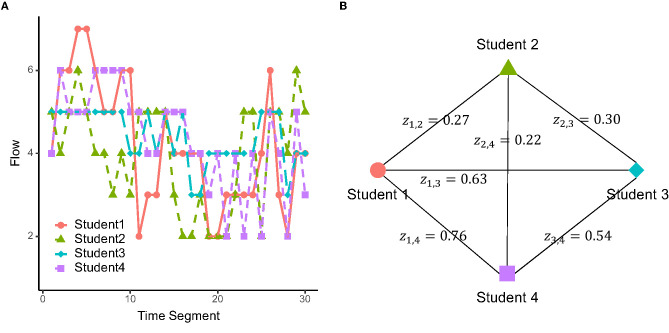
**(A)** Example of retrospectively rated flow dynamics in a group. **(B)** Flow dynamics similarity values between the members of the same group, evaluated by Fisher z-transformed temporal correlation.

Figure 2A shows an example of the flow dynamics for a group of four students. In this group, Student 2's flow dynamics were relatively divergent from the other members, with lower temporal correlation values with other members (*z*_1,2_ = 0.27, *z*_2,3_ = 0.30, *z*_2,4_ = 0.22), compared to the more convergent flow dynamics between the other three (*z*_1, 3_ = 0.63, *z*_1,4_ = 0.76, *z*_3,4_ = 0.54; Figure 2B).

**Figure 3:**
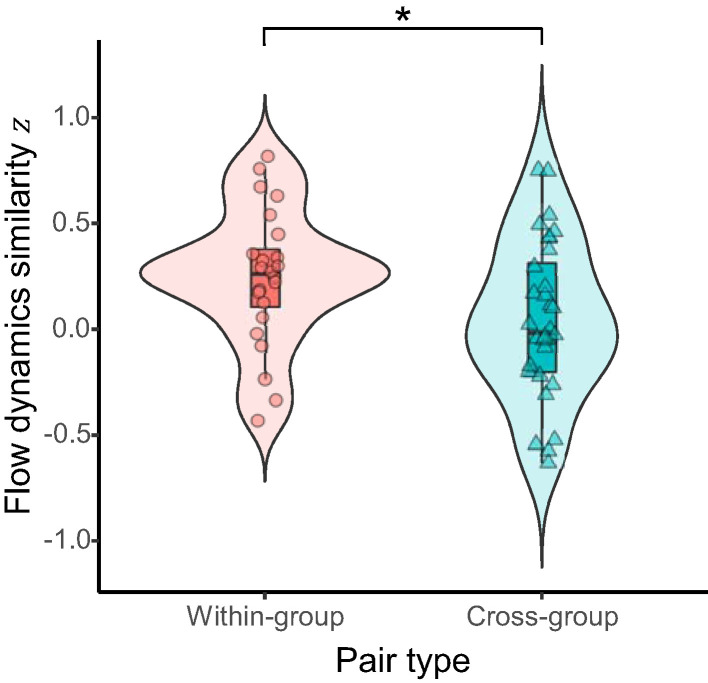
Similarity of flow dynamics between pairs of students within the same active-learning group (within-group) vs. across different groups (cross-group). Asterisk indicates significant difference (*p* < 0.05) between the pair types.

For hypothesis (1) that flow dynamics would be more convergent for the within-group pairs than for the cross-group pairs, we compared the similarity of flow dynamics between within-group and cross-group pairs. A permutation test showed that the temporal correlation of flow time series between the student pairs within the same learning group was significantly higher than that between the pairs of students assigned across different learning groups (difference of means = 0.204, 95% CI = 0.019–0.388, *p* = 0.036, Cohen's *d* = 0.59; Figure 3). This indicates that the flow dynamics were significantly more similar for members of the same group than for those in different groups.

### Inter-brain Synchronization Within and Between Groups

**Figure 4:**
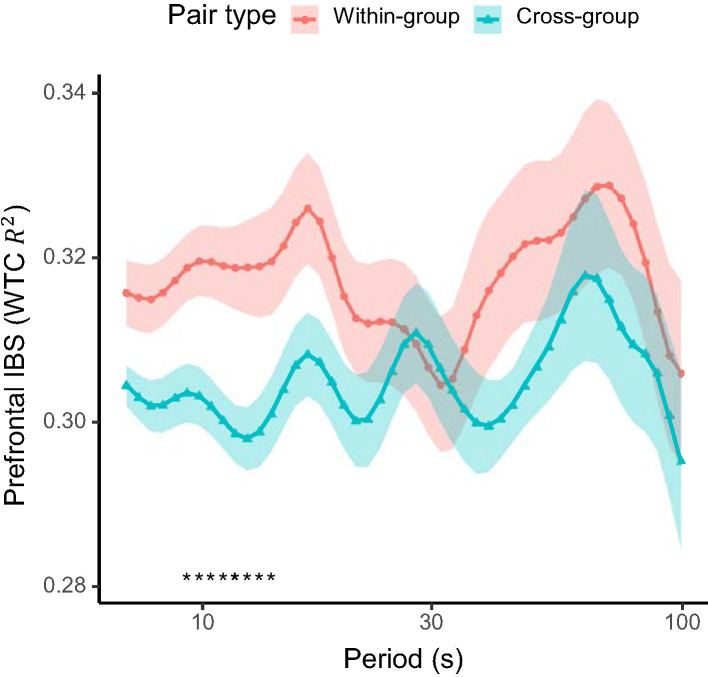
Prefrontal inter-brain synchronization (IBS) between pairs of students within the same active-learning group (within-group) and across different groups (cross-group). Solid lines show the mean and shaded areas the standard error of the mean for each pair type. Asterisks at the bottom indicates periods with a significant difference (q < 0.05; FDR-adjusted) between the pair types.

For hypothesis (2) that inter-brain synchronization would be higher for within-group pairs than for cross-group pairs, we compared the medial prefrontal inter-brain synchronization between the within-group and cross-group pairs. On average, a higher inter-brain synchronization was found between the members of a same groups over a wide range of time scales (Figure 4). Permutation tests at each Fourier period indicated that the inter-brain synchronization difference was significant in the range of periods 9.3–13.9 s (FDR-adjusted q < 0.05). This indicates that inter-brain synchronization across these time scales was sensitive to the collaborative interaction. The mean inter-brain synchronization in this period of interest was used in the subsequent correlation analyses.

**Figure 5:**
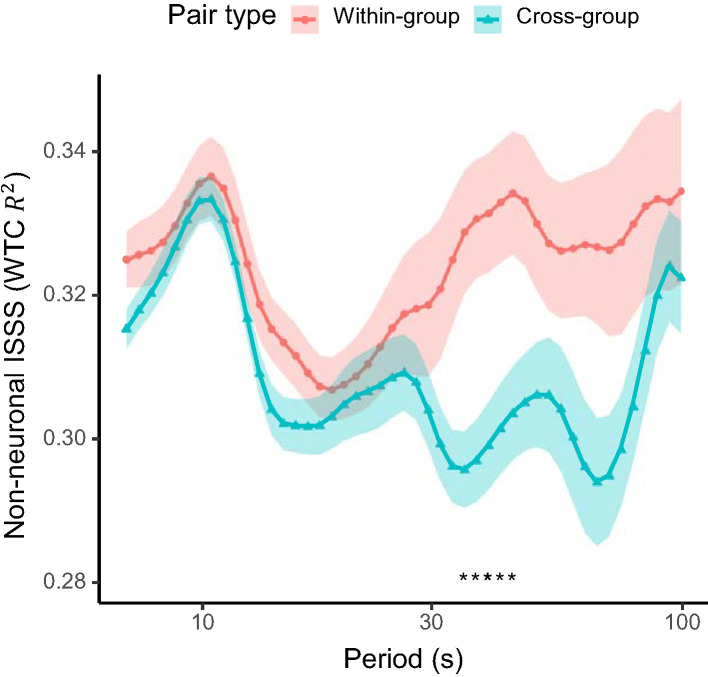
Non-neuronal interpersonal shallow signal synchronization (ISSS) between pairs of students within the same active-learning group (within-group) and across different groups (cross-group). Solid lines show the mean and shaded areas the standard error of the mean for each pair type. Asterisks at the bottom indicates periods with a significant difference (q < 0.05; FDR-adjusted) between the pair types.

In addition, we compared the non-neuronal interpersonal shallow signal synchronization from the 1 cm source-detector channels between the within-group and cross-group pairs. No significant differences were found in interpersonal shallow signal synchronization in the period of interest identified above, though some significant differences were observed across longer time scales (35.0–44.1 s; Figure 5).

### Correlation Between Flow Dynamics Similarity and Inter-brain Synchronization

**Figure 6:**
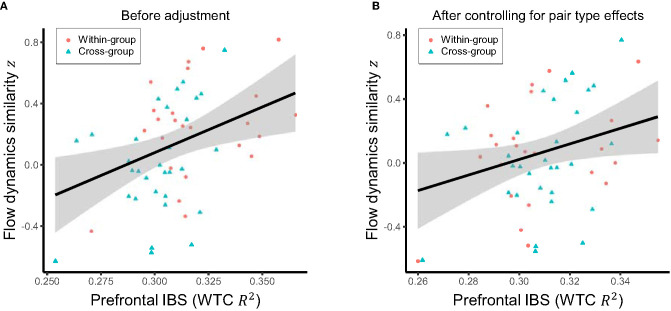
**(A)** Raw relationship between the mean inter-brain synchronization (IBS) in the period of interest (9.3–13.9 s) and the similarity of flow dynamics over the pairs. **(B)** Relationship between the mean IBS in the same period of interest and the similarity of flow dynamics after controlling for the effects of within-group vs. cross-group pair types. Solid line and shared area in each panel indicate the linear regression fit and its 95% level interval, respectively.

Regarding hypothesis (3) that higher prefrontal inter-brain synchronization is positively associated with more similar flow dynamics, we tested the correlation between mean inter-brain synchronization in the period of interest (9.3–13.9 s) and the similarity in flow dynamics amongst pairs of students. This revealed a significant positive correlation (*r* = 0.36, *p* = 0.004; Figure 6A). Since both the similarity of flow dynamics and the inter-brain synchronization were higher for the within-group than cross-group pairs, this correlation would have been at least partially mediated by the effects of pair types (within- vs. cross-group). Therefore, we also tested a modified version of hypothesis (3), which is orthogonal to hypotheses (1) and (2) by evaluating the partial correlation between flow dynamics similarity and inter-brain synchronization after controlling for the effects of pair types. The result remained significant (*r* = 0.28, *p* = 0.022; Figure 6B), indicating a direct positive association between inter-brain synchronization and flow dynamics similarity.

**Figure 7:**
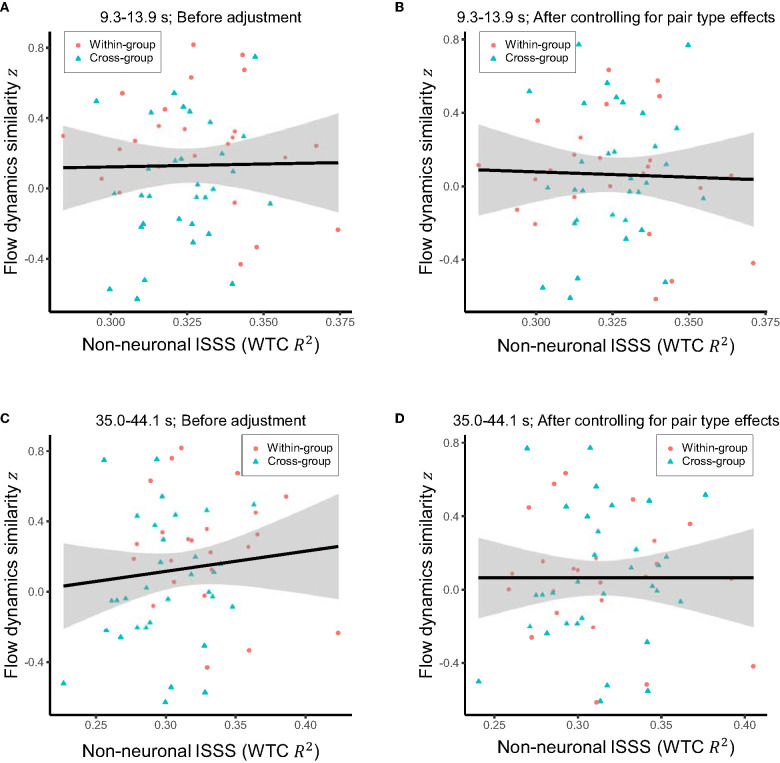
**(A)** Raw relationship between the mean interpersonal shallow signal synchronization (ISSS) in the period of interest (9.3–13.9 s) and the similarity of flow dynamics over the pairs. **(B)** Relationship between the mean ISSS in the same period of interest and the similarity of flow dynamics after controlling for the effects of within-group vs. cross-group pair types. **(C)** Raw relationship between the mean ISSS in the period range 35.0–44.1 s where ISSS was significantly higher for within-group than cross-group pairs and the similarity of flow dynamics over the pairs. **(D)** Relationship between the mean ISSS in the period range 35.0–44.1 s and the similarity of flow dynamics after controlling for the effects of pair types. Solid line and shared area in each panel indicate the linear regression fit and its 95% level interval, respectively. No significant correlations were observed.

Correlation analyses were repeated with the non-neuronal interpersonal shallow signal synchronization. There were no significant correlations with flow dynamics similarity (Figures 7A–D), supporting the neural origin of the observed association between inter-brain synchronization and shared flow dynamics.

## Discussion

In this study, we analyzed the similarity or sharing of fluctuating flow experiences and prefrontal inter-brain synchronization among students in real collaborative learning activities held in EFL classes in university education. In accordance with our hypotheses, we found that (1) flow dynamics were more convergent for the within-group than the cross-group pairs; (2) prefrontal inter-brain synchronization was higher among group members who directly collaborated than between cross-group learners who were just in the same class; and (3) prefrontal inter-brain synchronization was significantly positively correlated with the flow dynamics similarity, even after controlling for the effects of pair types. Below, we discuss the possible mechanisms and implications of these results, along with limitations and future directions.

### Shared Flow Dynamics in Collaboration

We observed that the temporal correlation of the flow state fluctuation was higher for the within-group pairs, indicating that collaborative learning enhanced the sharing of flow dynamics. Multiple and inter-related processes can lead to shared flow dynamics in collaborating groups. First, as participants do the group work together, they face objectively easy and difficult parts at the same time. This will lead to a temporal alignment of perceived challenges, especially among group members with similar skill levels. Furthermore, collaboration leads to the assimilation of effective skills and perceived challenges between members. For example, when a task is too difficult for a student to solve alone, help from other group members can work as a type of “scaffolding” and effectively boost the student's skill level to meet the challenge. In comparison, even when the task is too easy for one member, helping and guiding a peer who is having difficulties will pose additional challenges and make the moment less boring. This kind of mutual adjustment toward the challenge-skill balance can be a function of collaborative learning (Watanabe and Swain, 2007; Storch, 2009; Walker, 2010). In addition, group members share moments of perceived achievement, and interaction can also cause emotional contagion (Barsade, 2002), both of which lead to temporally aligned affective processes. Taken together, the shared flow dynamics in collaborative learning groups could be regarded as a signature of high integration and unity between group members.

We should note that the shared flow dynamics invoked in this study are based on the existence of temporal fluctuations in individual flow states and their similarity between learners. Thus, even if the average flow level of the two learners was high, the similarity of flow dynamics could have been low if their fluctuations were not temporally aligned. Our shared flow dynamics also overlap with the concept of “group flow.” However, the definition of group flow is not limited to sharing of individual flow and is more heterogeneous, with different emphasis on various individual and collective perspectives depending on the study context (Pels et al., 2018).

### Inter-brain Synchronization and Its Relation to Shared Flow Dynamics

Prefrontal inter-brain synchronization was also higher in the within-group than in the cross-group pairs. This result is consistent with previous hyperscanning studies that found both verbal and non-verbal cooperative interactions enhance prefrontal inter-brain synchronization between interacting individuals (Cui et al., 2012; Liu et al., 2016; Nozawa et al., 2016, 2019; Ikeda et al., 2017; Li et al., 2020; Sun et al., 2020). The underlying mechanisms for the enhanced inter-brain synchronization between group members may include the temporally aligned perception of challenge and achievement, which were discussed above as the likely processes underlying shared flow dynamics. In addition, temporal alignment of more fundamental cognitive processes during collaborative interactions, such as perception and understanding of each other's utterances, mentalizing each other's thoughts and intentions, and shared attention to tasks and salient events during the group activity, could also contribute to higher inter-brain synchronization within learning groups in high mutual engagement.

Among the time scales of low frequency oscillations (Zuo et al., 2010), the inter-brain synchronization differences between the within-group and cross-group pairs was significant in a relatively short time scale, overlapping with the so-called “slow-3” sub-band (0.073–0.198 Hz or 5.1–13.7 s), but not in the longer time scales. This is perhaps not surprising given that the same group work was carried out in parallel among the groups in the same class. Furthermore, all the students shared major cognitive events at longer time scales, such as the switch timings between group activities (GW1–4), general from-beginning-to-end progress pattern in each group work, and instructions and advice given by the teacher to the whole class. On the other hand, occurrences and timings of more detailed interaction events, challenges, and their solutions/achievements would have been more likely to vary among groups, leading to higher within-group inter-brain synchronization on a shorter time scale.

Finally, we observed that the prefrontal inter-brain synchronization at the time scale with sensitivity to the within-group collaborative interaction was significantly and positively correlated with the similarity of flow dynamics over the pairs. This remained true even after controlling for the mediating effects of the pair types on the two variables, and thus supports our hypothesis that convergence and divergence of flow dynamics between learners in collaborative tasks are reflected in the prefrontal inter-brain synchronization. From a neuroergonomic viewpoint, our results suggest a promising possibility of utilizing the prefrontal inter-brain synchronization for detection of an “isolated learner” in terms of their flow dynamics within a group (as exemplified in Figure 2). In addition, prefrontal inter-brain synchronization could be used for comparative and longitudinal evaluation of convergent/divergent flow dynamics on a group level. Such information could help teachers in deciding to take specific actions to facilitate flow, as well as learners to improve their manner of collaboration.

Additional analysis revealed that the interpersonal synchronization of the non-neuronal shallow signals was significantly higher for the within-group than for the cross-group pairs in the period range of 35.0–44.1 s. This time scale was longer than the period of interest (9.3–13.9 s) in which the significantly enhanced inter-brain synchronization for the within group was observed. A previous study on the frequency content of skin blood flow (Söderström et al., 2003) showed that sympathetic nerve control is involved in oscillations of skin blood flow signals in the overlapping time scale (“interval II”; 0.02–0.05 Hz or 20–50 s). Consistently, another study (Kirilina et al., 2013) identified a significant contribution of the scalp blood flow fluctuations in the forehead at the corresponding time scale (“A-band”; 0.02–0.04 Hz or 25–50 s) to the forehead fNIRS signals, and called for caution on the potential influence of sympathetic control. These suggest that the collaborative interaction induced interpersonal synchronization in sympathetic nerve regulation. On the other hand, the interpersonal shallow signal synchronization in either the shorter or longer time scales revealed no significant correlation with flow dynamics similarity. These results indicate that the sharing of flow dynamics is better captured by the interpersonal synchronization of the prefrontal cortical activities rather than that of the autonomic blood flow regulation processes.

### Limitations and Future Directions

The limitations of this study and possible future research directions should be noted. First, this study used a relatively small sample size and a specific activity design for collaborative learning. It is left for future studies to replicate the current results in a wider range of learning situations. In addition, we conducted the analyses using the student pair or dyad as a unit, because it is the basic unit whereby the sharing of flow dynamics and inter-brain synchronization can be defined. (Note that the statistical dependence structure in the dyadic data has been controlled for by the subject-based permutation procedures.) On the other hand, investigating the relationship between the inter-brain synchronization and shared flow dynamics at the group level would be interesting since students work as groups on collaborative learning. For this purpose, a larger sample with more groups or a repetition of multiple classroom sessions will be needed. Second, the fNIRS recording site in this study was limited to the mPFC. It will be meaningful to shed light on the relationship between the shared flow dynamics and the inter-brain synchronization in other cortical areas involved in social cognitions and interaction, such as the temporoparietal junction, superior temporal gyrus/sulcus, inferior frontal gyrus and so on. In addition, it would be more convincing to demonstrate that some areas contribute to the shared experience, whereas other areas do not. Next, although the degree of subjective experience sharing and inter-brain synchronization were correlated, this does not prove that inter-brain synchronization is the mechanism behind a shared flow experience. To understand the dynamic process of sharing flow and to determine how much significant role inter-brain synchronization plays in the process as “hidden variables” that cannot be observed externally (Kingsbury and Hong, 2020), simultaneous measurement of behavioral interactions between learners as well as individual systemic physiological activities could help. This can lead to the further endeavor of exploring how informative those peripheral signals could be in predicting flow and its sharing, and how such information could be overlapping with or independent from the information conveyed by inter-brain synchronization. (The video recordings for retrospective rating of flow dynamics in this study were limited and uneven in terms of the view coverage and directions for the target group members, thus not adequate for such behavioral analysis.) Another remaining issue is the impact of shared flow on various outcomes in group learning. Recent studies have begun to elucidate how inter-brain synchronization captured by EEG and fNIRS hyperscanning correlates with better team performance in several settings, including visual search tasks (Szymanski et al., 2017), collaborative problem solving (Antonenko et al., 2019; Reinero et al., 2021), and learning success in teacher-student settings (Holper et al., 2013; Pan et al., 2018, 2020; Liu et al., 2019). However, it remains to be confirmed whether shared experiences with peers, as marked by the prefrontal inter-brain synchronization, can inform success or failure in a learner achieving their goals.

Despite the above limitations, to the best of our knowledge, this is the first study to demonstrate that prefrontal inter-brain synchronization during collaborative learning can be an objective marker for the convergence and divergence of flow dynamics between learners. In the future, prefrontal inter-brain synchronization could be used to evaluate the convergent engagement of students in active group learning tasks, with proper feedback provided to the teacher or the students themselves to facilitate further collaboration. The technology may also be applicable to the detection of left-behind students in lecture-style learning settings as well as in the promotion of a wider range of collaborative activities. We are hoping to extend this study toward such practical neuroergonomic applications in the field of education. This pursuit will be benefited from and contribute to the wide effort to overcome the grand challenges for neuroergonomics in general, such as the improvement and combining of sensors, incorporation of advanced analytical methods and artificial intelligence approaches, development of design principles and techniques for better user experience, and consensus building for privacy and ethical issues (Dehais et al., 2020; Fairclough and Lotte, 2020).

## Data Availability Statement

The data that support the findings of this study are available from the corresponding author upon reasonable request.

## Ethics Statement

The studies involving human participants were reviewed and approved by Ethics Committee of Tohoku University Graduate School of Medicine. The patients/participants provided their written informed consent to participate in this study.

## Author Contributions

TN contributed to the study design, experimentation, analysis, and manuscript preparation. MK contributed to the study design, experimentation, organization, and instruction in the EFL classes. RY and YI contributed to the study design, experimentation, and preparation of the EFL classes. HJ, SI, and KS contributed to the study design and experimentation. YM contributed to the interpretation of the results. RK contributed to the supervision and provided useful discussions throughout the project. All authors contributed to manuscript revision, read, and approved the submitted version.

## Conflict of Interest

The authors declare that the research was conducted in the absence of any commercial or financial relationships that could be construed as a potential conflict of interest.
